# 4482 Horizontal ridge augmentation in maxilla using bone expansion vs bone splitting techniques in adult patients: a prospective cohort

**DOI:** 10.1017/cts.2020.335

**Published:** 2020-07-29

**Authors:** Amanda Figueroa Acevedo, José E. Pedroza, Augusto Elias-Boneta, Elizabeth Orsini, Lidia M. Guerrero, Sona Rivas-Tumanyan

**Affiliations:** 1 University of Puerto Rico, Medical Sciences Campus

## Abstract

OBJECTIVES/GOALS: The objective of the study is to compare two horizontal bone augmentation techniques (bone expansion and bone splitting) that are currently used for horizontally deficient maxillary ridges. Bone expanded in millimeters (mm), implant stability, and patient satisfaction will be compared with each technique. METHODS/STUDY POPULATION: This pilot (prospective cohort) study will be divided in two sites, a private practice and the Oral and Maxillofacial Surgery (OMS) Clinic at the University of Puerto Rico, School of Dental Medicine. A total of 20 patients will be selected, 10 patients in each site. In both sites, pre-operative and post-operative Cone Beam CT radiographs will be taken to measure bone width. Implant stability will be measured using an Osstell. 2 weeks post-surgery, a patient satisfaction questionnaire will be given to patients. A two-sample T test will be used to compare techniques statistically. RESULTS/ANTICIPATED RESULTS: We anticipate that bone expansion will be as good as (non-inferiority) bone splitting in terms of bone expanded in millimeters to desire width, and implant diameter will not be compromised. We also expect that implants placed with the bone manipulation technique will have a higher implant stability at baseline and less pain, discomfort and swelling in terms of patient satisfaction. DISCUSSION/SIGNIFICANCE OF IMPACT: Our contributions here are expected to illustrate clinical and radiographic bone expansion techniques that will enhance implant placement treatment for implantologists and patient’s experience.

